# Generative Artificial Intelligence Performs at a Second-Year Orthopedic Resident Level

**DOI:** 10.7759/cureus.56104

**Published:** 2024-03-13

**Authors:** Zachary C Lum, Dylon P Collins, Stanley Dennison, Lohitha Guntupalli, Soham Choudhary, Augustine M Saiz, Robert L Randall

**Affiliations:** 1 Orthopedic Surgery, University of California (UC) Davis School of Medicine, Sacramento, USA; 2 Orthopedic Surgery, Nova Southeastern University, Pembroke Pines, USA; 3 College of Medicine, Nova Southeastern University Dr. Kiran C. Patel College of Osteopathic Medicine, Fort Lauderdale, USA; 4 Osteopathic Medicine, Nova Southeastern University Dr. Kiran C. Patel College of Osteopathic Medicine, Clearwater, USA; 5 Orthopedic Surgery, University of California, Davis, Davis, USA; 6 Orthopedic Surgery, University of California (UC) Davis Health, Sacramento, USA

**Keywords:** oite, generative artificial intelligence, orthopaedic surgery, google bard, chatgpt

## Abstract

Introduction

Artificial intelligence (AI) models using large language models (LLMs) and non-specific domains have gained attention for their innovative information processing. As AI advances, it's essential to regularly evaluate these tools' competency to maintain high standards, prevent errors or biases, and avoid flawed reasoning or misinformation that could harm patients or spread inaccuracies. Our study aimed to determine the performance of Chat Generative Pre-trained Transformer (ChatGPT) by OpenAI and Google BARD (BARD) in orthopedic surgery, assess performance based on question types, contrast performance between different AIs and compare AI performance to orthopedic residents.

Methods

We administered ChatGPT and BARD 757 Orthopedic In-Training Examination (OITE) questions. After excluding image-related questions, the AIs answered 390 multiple choice questions, all categorized within 10 sub-specialties (basic science, trauma, sports medicine, spine, hip and knee, pediatrics, oncology, shoulder and elbow, hand, and food and ankle) and three taxonomy classes (recall, interpretation, and application of knowledge). Statistical analysis was performed to analyze the number of questions answered correctly by each AI model, the performance returned by each AI model within the categorized question sub-specialty designation, and the performance of each AI model in comparison to the results returned by orthopedic residents classified by their respective post-graduate year (PGY) level.

Results

BARD answered more overall questions correctly (58% vs 54%, p<0.001). ChatGPT performed better in sports medicine and basic science and worse in hand surgery, while BARD performed better in basic science (p<0.05). The AIs performed better in recall questions compared to the application of knowledge (p<0.05). Based on previous data, it ranked in the 42nd-96th percentile for post-graduate year ones (PGY1s), 27th-58th for PGY2s, 3rd-29th for PGY3s, 1st-21st for PGY4s, and 1st-17th for PGY5s.

Discussion

ChatGPT excelled in sports medicine but fell short in hand surgery, while both AIs performed well in the basic science sub-specialty but performed poorly in the application of knowledge-based taxonomy questions. BARD performed better than ChatGPT overall. Although the AI reached the second-year PGY orthopedic resident level, it fell short of passing the American Board of Orthopedic Surgery (ABOS). Its strengths in recall-based inquiries highlight its potential as an orthopedic learning and educational tool.

## Introduction

In recent years, the fields of machine learning, deep learning, and artificial intelligence (AI) have seen exceptional growth, revolutionizing various sectors such as manufacturing, consumer products, and healthcare. Neural networks, in particular, have advanced the detection of fractures and orthopedic implants, among other medical applications [1-6]. Nevertheless, these AI systems are often domain-specific, requiring significant time, resources, and specialized data for their respective fields, which limits their broad applicability and versatility.

Large language models (LLMs) present an alternative approach to machine learning by leveraging vast amounts of data to generate responses that are more akin to natural language [7]. Operating in non-domain-specific or few-shot contexts, they need minimal data to perform specific tasks. LLMs have the potential to understand, process, analyze, and reason through a diverse array of questions. Recently, two AI models named Chat Generative Pre-trained Transformer (ChatGPT) and BARD, which utilize LLMs in non-specific domain areas, have attracted considerable attention.

Medical education and technology are experiencing a transformation with the emerging application of AI through computer-based models, virtual reality simulations, and tailored learning platforms [8,9]. With the expanding capabilities of AI, it is imperative to consistently evaluate the competence of AI-powered tools, especially generative AI models that can generate flawed reasoning or misinformation. Recognizing the public availability of these tools and their utility to serve as additional informational aids makes verifying the accuracy of these tools crucial, especially within the field of orthopedic surgery, as mistakes could be detrimental to patients or lead to the spread of misinformation.

The premise of this study was to explore the percentage of Orthopedic In-Training Examination (OITE) questions the generative, pre-trained transformer chatbots, ChatGPT and BARD, could correctly answer. Second, the study investigated whether or not AI performance varied depending on the sub-specialty subject matter or the taxonomy of the questions (recall, interpretation, and application of knowledge). Third, the study compared the performance of both LLMs to one another. Lastly, the study investigated how the performance of the LLMs stood up against that of orthopedic residents at various training levels, particularly focusing on the likelihood of the LLMs to yield a passing score on the orthopedic surgery written boards, for which the benchmark of the 10th percentile for fifth-year residents is typically considered a passing score.

## Materials and methods

In this experimental study, we used commercially available LLMs named ChatGPT 3.5 (OpenAI, San Francisco, CA) and BARD (Alphabet Inc, Mountain View, CA), which incorporate self-attention mechanisms and a vast array of training data to produce natural language responses in conversational contexts. These models excel at managing long-range dependencies in text, resulting in coherent and contextually relevant responses. Self-attention mechanisms are critical in natural language processing tasks such as language translation and text generation, helping to discern the relationships between words or elements within sentences or entire documents. The synergy of long-range dependencies and self-attention enables the models to understand and generate accurate responses. ChatGPT 3.5 operates as a closed system, confined to a server without internet access, and relies on intrinsic word relationships within its neural network to generate responses. This differentiates it from other chatbots or domain-specific AI that utilize internet-based searches. Conversely, BARD operates similarly but is permitted internet access, potentially enhancing its informational reach.

We selected 757 questions from the actual OITE from the years 2015-2016 and 2022. The 2022 exam served as a benchmark since its questions and answers were not included in the training dataset. We excluded 48% (367 of 757) of the questions because they incorporated images, figures, tables, or charts, leaving 390 questions for BARD. Additionally, three questions were removed from ChatGPT’s set due to the AI's inability to provide a definitive answer, resulting in 387 questions for ChatGPT. As ChatGPT is a text-only program, it cannot process questions with non-textual data such as images or figures. We entered each question into ChatGPT’s interface in separate chat sessions to prevent any memory retention, which could occur through the LLM’s recurrent neural network learning processes.

For evaluation, we entered each question into the chat session and requested the LLM to select an answer. If the LLM failed to choose a single answer or provided multiple answers, we re-prompted with “Select the single best answer.” If the LLM still failed to select one, we recorded the question as "did not answer." ChatGPT struggled with 0.7% (three of 390) of the questions in providing a single best answer, which we then excluded. In contrast, BARD managed to respond to all its applicable questions.

Primary and secondary study outcomes

The primary goal was to determine the percentage of questions each LLM accurately answered. Secondary aims included a detailed comparison of performance across ten sub-specialties and three taxonomy classes of questions, benchmarking against orthopedic residents' training levels, and evaluating against a pass rate threshold for the American Board of Orthopedic Surgery (ABOS).

We employed the Buckwalter taxonomic schema to classify question difficulty levels [10]. Among the 757 questions, 62% (242 of 390) were Tax I (recognition and recall), 13% (52 of 390) were Tax II (comprehension and interpretation), and 25% (96 of 390) were Tax III (application of knowledge).

We used the mean and standard deviation of OITE scores by year and post-graduate year (PGY) level to compare the LLMs to orthopedic residents. This included analyzing mean scores, standard deviations, and calculated percentiles for each PGY level [11]. We also assessed the likelihood of the LLMs passing the ABOS written exam based on a correlation between OITE scores in the 10th percentile and ABOS exam failure rates [12].

Ethical approval

Since the study did not include human or animal participants, ethics committee approval was not obtained.

Statistical analysis

We applied chi-squared tests to assess performance differences between the LLMs. ANOVA contrast deviation was used to evaluate the variance between each sub-specialty and the cohort average. We used Omnibus likelihood ratio tests to detect response correctness concerning question subject types and taxonomy classes. If differences were found, binomial logistic regression tests compared the correct and incorrect answers within these categories. Estimated marginal means were computed with 95% confidence intervals, and visual representations were created for both subject types and taxonomy classes. All statistical analyses were performed using Jamovi software version 2.3.21.0 (Sydney, Australia). 

## Results

Percentage of OITE questions answered correctly

ChatGPT correctly answered 54% (210 out of 387) of the questions and incorrectly answered 46% (177 out of 387). Three questions received no response from the AI; these were excluded because they elicited multiple answers without a clear "single best answer."

BARD correctly answered 58% (227 out of 390) of the questions and incorrectly answered 42% (163 out of 390), responding to all posed questions.

Performance in relation to sub-specialty knowledge

ChatGPT’s performance varied by sub-specialty. It performed best in sports medicine (73%, 27 of 37) and worst in hand surgery (28%, nine of 32). ANOVA analysis showed sports medicine and basic science scores above average (p=0.006 and p=0.009, respectively), while hand surgery was below average (p=0.007) (Tables 1, 2).

**Table 1:** All questions (OITE + SAE) were answered by ChatGPT and BARD based upon subject type. The estimated margin means with 95% confidence intervals are displayed. BS: basic science, TR: trauma, SM: sports medicine, SP: spine, HK: hip and knee reconstruction, PE: pediatrics, OC: oncology, SE: shoulder and elbow, HA: hand surgery, FA: foot and ankle, AN: anatomy, Q: question, CI: confidence interval, ANOVA: analysis of variance, OITE: orthopedic in-training examination, SAE: self-assessment exam, ChatGPT: Chat Generative Pre-trained Transformer.

Subject	Total Q	Correct	% Correct	Estimated margin means (95% CI)	Total Q	Correct	% Correct	Estimated margin means (95% CI)
	ChatGPT				BARD			
BS	114	75	65.8	0.66 (0.56-0.73)	117	83	70.9	0.71 (0.63-0.79)
TR	27	63	42.9	0.43 (0.31-0.55)	62	37	59.7	0.60 (0.42-0.66)
SM	27	37	73.0	0.73 (0.57-0.84)	36	22	61.1	0.61 (0.47-0.78)
SP	11	19	57.9	0.58 (0.36-0.77)	20	11	55.0	0.55 (0.38-0.79)
HK	17	19	47.2	0.47 (0.32-0.63)	36	20	55.6	0.56 (0.30-0.62)
PE	17	11	60.7	0.61 (0.48-0.80)	32	22	68.8	0.69 (0.53-0.81)
OC	9	9	50.0	0.50 (0.28-0.72)	17	5	29.4	0.29 (0.12-0.54)
SE	13	13	50.0	0.50 (0.32-0.68)	26	11	42.3	0.42 (0.25-0.62)
HA	9	23	28.1	0.28 (0.16-0.47)	31	11	35.5	0.36 (0.20-0.52)
FA	5	9	35.7	0.36 (0.16-0.62)	13	5	38.5	0.39 (0.21-0.68)
Total	387	210	54.3		390	227	58.2	

**Table 2:** Analysis of variance performed for ChatGPT sub-specialty question types. Contrasts show differences between variables and their group means, specifically that basic science and sports medicine performed better, while hand surgery performed worse than group averages (p<0.05). Pediatrics trended towards performing better (p=0.089). BS: basic science, TR: trauma, SM: sports medicine, SP: spine, HK: hip and knee reconstruction, PE: pediatrics, OC: oncology, SE: shoulder and elbow, HA: hand surgery, FA: foot and ankle, AN: anatomy, SE: standard error, t: t-value for analysis of variance contrasts, ChatGPT: Chat Generative Pre-trained Transformer.

Contrasts-sub-specialty	Estimate	SE	t	p
TR-FA, TR, PE, OC, HK, SP, HA, SM, SE, BS	−0.0874	0.0622	−1.405	0.161
PE-FA, TR, PE, OC, HK, SP, HA, SM, SE, BS	0.1403	0.0824	1.702	0.089
OC-FA, TR, PE, OC, HK, SP, HA, SM, SE, BS	−0.0160	0.1068	−0.150	0.881
HK-FA, TR, PE, OC, HK, SP, HA, SM, SE, BS	−0.0438	0.0783	−0.559	0.577
SP-FA, TR, PE, OC, HK, SP, HA, SM, SE, BS	0.0630	0.1042	0.605	0.546
HA-FA, TR, PE, OC, HK, SP, HA, SM, SE, BS	−0.2257	0.0836	−2.701	0.007
SM-FA, TR, PE, OC, HK, SP, HA, SM, SE, BS	0.2138	0.0774	2.762	0.006
SE-FA, TR, PE, OC, HK, SP, HA, SM, SE, BS	0.0160	0.0903	-0.177	0.860
BS-FA, TR, PE, OC, HK, SP, HA, SM, SE, BS	0.1306	0.0499	2.615	0.009

BARD’s performance was more consistent across sub-specialties, with the highest scores in basic science (71%, 83 of 117) and the lowest in oncology (29%, five of 17). ANOVA indicated basic science above average (p<0.001), with sports medicine, hand surgery, and oncology near average (p=0.076, p=0.057, and p=0.059, respectively) (Table 3).

**Table 3:** Analysis of variance performed for BARD sub-specialty question types. Contrast deviation shows basic science performed better than means, with sports medicine trending towards higher performance (p<0.001, p=0.076, respectively). While no sub-specialty performed significantly worse, both oncology and hand surgery trended towards worse performance (p=0.059, p=0.057, respectively). BS: basic science, TR: trauma, SM: sports medicine, SP: spine, HK: hip and knee reconstruction, PE: pediatrics, OC: oncology, SE: shoulder and elbow, HA: hand surgery, FA: foot and ankle, AN: anatomy, SE: standard error, t: t-value for analysis of variance contrasts.

Contrasts–sub-specialty	Estimate	SE	t	p
TR-FA, TR, PE, OC, HK, SP, HA, SM, SE, BS	0.0405	0.0627	0.647	0.518
PE-FA, TR, PE, OC, HK, SP, HA, SM, SE, BS	0.0620	0.0820	0.757	0.450
OC-FA, TR, PE, OC, HK, SP, HA, SM, SE, BS	−﻿0.2063	0.1091	−1.892	0.059
HK-FA, TR, PE, OC, HK, SP, HA, SM, SE, BS	−0.0433	0.0788	−0.549	0.583
SP-FA, TR, PE, OC, HK, SP, HA, SM, SE, BS	0.0995	0.1012	0.984	0.326
HA-FA, TR, PE, OC, HK, SP, HA, SM, SE, BS	−0.1567	0.0820	−1.912	0.057
SM-FA, TR, PE, OC, HK, SP, HA, SM, SE, BS	0.1384	0.0779	1.777	0.076
SE-FA, TR, PE, OC, HK, SP, HA, SM, SE, BS	−0.0774	0.0898	﻿−0.861	0.390
BS-FA, TR, PE, OC, HK, SP, HA, SM, SE, BS	0.2151	0.0497	﻿ 4.328	<0.001

Performance in relation to increasingly difficult taxonomic level

ChatGPT correctly answered 59% of Taxonomy 1 questions, 58% of Taxonomy 2, and 39% of Taxonomy 3. Binomial logistic regression indicated Taxonomy 1 questions had higher accuracy than Taxonomy 3 (p=0.043) (Table 4).

**Table 4:** Binomial logistic regression based upon 387 taxonomy questions. ChatGPT exhibited a higher likelihood of correctly answering a recognition and recall question (Taxonomy 1) versus application of knowledge question (Taxonomy 3) (p=0.043, 1.729 OR (1.017-2.939)). There were no differences between interpretation questions (Taxonomy 2) and recognition and recall questions (p=0.994). BS: basic science, TR: trauma, SM: sports medicine, SP: spine, HK: hip and knee reconstruction, PE: pediatrics, OC: oncology, SE: shoulder and elbow, HA: hand surgery, FA: foot and ankle, AN: anatomy, SE: standard error, z: z-score, ChatGPT: Chat Generative Pre-trained Transformer.

Model coefficients-ChatGPT						95% confidence interval	
Predictor	Estimate	SE	Z	p	Odds ratio	Lower	Upper
Intercept	−0.62419	0.199	−3.14301	0.002	0.536	0.363	0.791
Taxonomy							
2–1	0.00259	0.326	0.00792	0.994	1.003	0.529	1.901
3–1	0.54772	0.271	2.02367	0.043	1.729	1.017	2.939
Sub-specialty							
FA-BS	0.94869	0.606	1.56628	0.117	2.582	0.788	8.464
TR-BS	0.74335	0.329	2.25656	0.024	2.103	1.103	4.011
PE-BS	−0.25623	0.438	−0.58538	0.558	0.774	0.328	1.825
OC-BS	0.44234	0.520	0.85144	0.395	1.556	0.562	4.309
HK-BS	0.57065	0.395	1.44571	0.148	1.769	0.816	3.835
SP-BS	0.24643	0.506	0.48706	0.626	1.279	0.475	3.449
HA-BS	1.30327	0.456	2.85583	0.004	3.681	1.505	9.004
SM-BS	−0.56171	0.431	−1.30445	0.192	0.570	0.245	1.326
SE-BS	0.41388	0.452	0.91667	0.359	1.513	0.624	3.665

BARD correctly answered 63% of Taxonomy 1 questions, 58% of Taxonomy 2, and 47% of Taxonomy 3. Binomial logistic regression showed Taxonomy 1 outperformed Taxonomy 3 (p=0.035) (Table 5).

**Table 5:** BARD performed better in Taxonomy type 1 questions than Taxonomy type 3 by binomial log regression (p=0.035, OR 1.767 (1.041-2.998)). There were no differences between interpretation questions (Taxonomy 2) and recognition and recall questions (p=0.676). BS: basic science, TR: trauma, SM: sports medicine, SP: spine, HK: hip and knee reconstruction, PE: pediatrics, OC: oncology, SE: shoulder and elbow, HA: hand surgery, FA: foot and ankle, AN: anatomy, SE: standard error, z: z-score.

Model coefficients-BARD						95% Confidence Interval	
Predictor	Estimate	SE	Z	p	Odds ratio	Lower	Upper
Intercept	−0.961	0.211	−4.555	<0.001	0.383	0.253	0.579
Taxonomy							
2–1	0.135	0.323	0.418	0.676	1.144	0.608	2.154
3–1	0.569	0.270	2.110	0.035	1.767	1.041	2.998
Sub-specialty							
FA-BS	0.959	0.594	1.615	0.106	2.610	0.815	8.364
TR-BS	0.602	0.339	1.775	0.076	1.825	0.939	3.548
PE-BS	0.453	0.427	1.060	0.289	1.573	0.681	3.636
OC-BS	1.666	0.576	2.891	0.004	5.293	1.710	16.380
HK-BS	0.958	0.404	2.373	0.018	2.607	1.181	5.753
SP-BS	0.476	0.503	0.947	0.344	1.610	0.601	4.312
HA-BS	1.340	0.442	3.033	0.002	3.817	1.606	9.072
SM-BS	0.157	0.417	0.376	0.707	1.170	0.516	2.650
SE-BS	1.037	0.459	2.259	0.024	2.821	1.147	6.937

Performance comparison with orthopedic residents

ChatGPT's performance ranked between the 42nd-95th percentile for PGY1s and between the 1st-7th for PGY5s across different OITE years. However, it likely would not pass the ABOS examination based on the PGY5 10th percentile benchmark (Table 6) [11,12].

BARD performed slightly better, ranking between the 61st-96th percentile for PGY1s and between the 1st-17th for PGY5s. Despite occasionally surpassing the 10th percentile mark, overall performance suggested it also would likely not pass the ABOS examination (Table 6) [11,12].

**Table 6:** OITE individual and combined percentile ranking. This table presents the percentile rank for each post-graduate year (PGY). The OITE provides specific mean raw scores and standard deviations for each PGY, enabling the calculation of percentiles for OITE 2015, 2016, and 2022. Based on previous OITE years (2014-2017), a mean raw score and standard deviation can be applied to non-specific OITE questions, such as those from AAOS SAE and all combined questions in testing, as shown below. OITE: orthopedic in-training examination, PGY: post-graduate year, AAOS: American Academy of Orthopedic Surgeons, SAE: self-assessment test.

ChatGPT	OITE 2015	OITE 2016	OITE 2022
PGY1	95th	79th	42nd
PGY2	50th	27th	30th
PGY3	13th	3rd	14th
PGY4	4th	1st	9th
PGY5	1st	1st	7th
BARD	OITE 2015	OITE 2016	OITE 2022
PGY1	96th	90th	61st
PGY2	54th	46th	47th
PGY3	15th	9th	29th
PGY4	5th	2nd	21st
PGY5	2nd	1st	17th

Performance comparison between ChatGPT and BARD

BARD had a higher percentage of correct answers than ChatGPT (58% vs 54%, p<0.001).

## Discussion

AI has become increasingly prevalent in medicine over the past few years, with potential applications in education, interpretation, and information management expanding [4]. Furthermore, it may ultimately enhance our precision in an array of sub-specialty diagnostics and therapeutics [13]. As new AI tools are developed, it is essential to assess, evaluate, and update their competency. In our study, ChatGPT, an AI LLM chatbot, correctly answered 54% of the questions on modern OITE-style exams, and BARD answered 58% correctly. While this places both AIs within the average percentile for a second-year orthopedic resident, it is unlikely to pass the ABOS due to its performance below the 10th percentile of upper-level residents. This result may be attributed to the chatbots' limited ability to apply knowledge to higher taxonomic-level questions, suggesting a difficulty in utilizing their knowledge in practical ways. This suggests that the model may have limitations in terms of its ability to integrate, synthesize, generalize, and apply factual knowledge in more nuanced ways. Furthermore, the AI would likely struggle to pass the ABOS due to its inability to interpret and analyze image-based questions, which make up roughly half of the test questions.

There are likely practical benefits and applications of AI in this context. One advantage of AI is its ability to manage large volumes of data, which can be quickly accessed as knowledge by users. This study demonstrated that the AI LLM performed better in recognition, recall, comprehension, and interpretation tasks than in problem-solving and knowledge application. This lack of application of knowledge has been highlighted before in previous publications [14]. Interestingly, in another study, this difference with regard to hierarchical question type was not seen with dermatology knowledge questions [15]. Other research has shown opportunities for AI to use big data for insights and strategies in managing specific diseases, such as opioid use disorders [4]. For example, Liu et al. found that AI and orthopedic surgeons had similar accuracy in identifying tibial plateau fractures [16]. These applications could enhance efficiency and precision in diagnosis and treatment, ultimately improving patient outcomes.

AI can also make educational resources more accessible to patients. A recent study showed that ChatGPT successfully revised complex patient education materials on spine surgery and joint replacement, making them readable at fifth- to sixth-grade levels [17]. Another study proposed that AI could enable educators to transition to mentorship roles by compiling the best learning strategies from top educators, allowing students to enhance their learning experiences independently and efficiently [18]. Furthermore, AI can offer personalized learning experiences tailored to individual students' needs and abilities, potentially improving engagement and knowledge retention for more effective learning. However, more research is needed to determine the extent and degree of these benefits.

This study has several limitations, particularly the inability to incorporate visual identification, interpretation, and integration within the questions. Almost half of the questions contained images, figures, or charts, leading to their exclusion. The actual ABOS and OITE exams include images, and many aspects of musculoskeletal care necessitate interpreting and analyzing images, radiographs, and tactile feedback from physical examinations. The exclusion of image-based questions may have biased the results by potentially omitting more challenging or application-focused questions for the LLM. Moreover, the basic science sub-specialty contained more recall-based questions, which could have inflated the LLM's performance in that area.

Although images play a crucial role in orthopedic surgery, this LLM relies solely on text input. While AI for image analysis is advancing rapidly, future iterations may be able to assess images. Nonetheless, this preliminary study of text-based questions was sufficient to reveal the LLM's capabilities and limitations in this context. General limitations of AI models include potential biases or inaccuracies in the datasets they are trained on, which can reflect or amplify existing societal biases or inequalities and may contain outdated information.

Lastly, limitations specific to this LLM stem from its training on broad, non-specific information. While it excels in summarization, translation, and text generation, it might struggle with context or nuanced language in specialized knowledge areas, leading to inaccurate or misleading responses.

## Conclusions

Though ChatGPT and BARD might not pass the ABOS written exam at this point, they offered well-structured explanations for correct answers, achieving results comparable to around the 50th percentile of PGY2 orthopedic residents. Furthermore, the model demonstrated learning capabilities when incorrect answers were corrected, as it retained and consistently applied the corrected information throughout the chat session. Overall, the ability to return well-structured, insightful explanations (to correctly answer questions) combined with demonstrated learning capabilities suggest AI's potential to support and enhance medical education and healthcare in the future.

The LLM exhibited strengths in recalling facts but faced challenges in applying knowledge. As AI technology advances, particularly in areas like image-based recognition, interpretation, and domain-specific knowledge application, it will be fascinating to observe the ongoing improvements in AI and explore its optimal application in orthopedic education.
